# Giant rotating magnetocaloric effect induced by highly texturing in polycrystalline DyNiSi compound

**DOI:** 10.1038/srep11929

**Published:** 2015-07-10

**Authors:** Hu Zhang, YaWei Li, Enke Liu, YaJiao Ke, JinLing Jin, Yi Long, BaoGen Shen

**Affiliations:** 1 School of Materials Science and Engineering, University of Science and Technology of Beijing, Beijing 100083, P. R. China; 2 State Key Laboratory for Magnetism, Institute of Physics, Chinese Academy of Sciences, Beijing 100190, P. R. China

## Abstract

Large rotating magnetocaloric effect (MCE) has been observed in some single crystals due to strong magnetocrystalline anisotropy. By utilizing the rotating MCE, a new type of rotary magnetic refrigerator can be constructed, which could be more simplified and efficient than the conventional one. However, compared with polycrystalline materials, the high cost and complexity of preparation for single crystals hinder the development of this novel magnetic refrigeration technology. For the first time, here we observe giant rotating MCE in textured DyNiSi polycrystalline material, which is larger than those of most rotating magnetic refrigerants reported so far. This result suggests that DyNiSi compound could be attractive candidate of magnetic refrigerants for novel rotary magnetic refrigerator. By considering the influence of demagnetization effect on MCE, the origin of large rotating MCE in textured DyNiSi is attributed to the coexistence of strong magnetocrystalline anisotropy and highly preferred orientation. Our study on textured DyNiSi not only provides a new magnetic refrigerant with large rotating MCE for low temperature magnetic refrigeration, but also opens a new way to exploit magnetic refrigeration materials with large rotating MCE, which will be highly beneficial to the development of rotating magnetic refrigeration technology.

Magnetocaloric effect (MCE) is a magneto-thermodynamic phenomenon in which a temperature change in magnetic material is caused by the variation of external magnetic field^1^,^2^. With the environmental degradation and energy shortage, magnetic refrigeration based on MCE has attracted considerable attention due to its energy efficiency and environment friendly in comparison with the conventional gas compression-expansion refrigeration^3^,^4^,^5^,^6^. In order to improve the applications of magnetic refrigeration technology, it is very important to search for materials with large MCE. Usually, the MCE is mainly contributed by the changes of exchange energy, magnetic anisotropy energy, and magnetoelastic energy^7^. Over the last few decades, a lot of studies have been made to investigate the contribution from the changes of exchange energy, i.e., the MCE related to the magnetic ordering change during the phase transition^8^,^9^,^10^. However, the contribution from magnetic anisotropy has not been sufficiently studied since it was considered to be much lower than the contribution from the ordering change^7^.

Very recently, some single crystals, such as 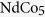
7, 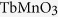
11, 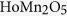
12, and 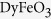
13, have been reported to exhibit large rotating MCE due to the strong magnetocrystalline anisotropy, opening up new research direction for magnetic refrigeration. Furthermore, a novel rotary magnetic refrigerator, in which a large MCE can be obtained by simply rotating the magnetic refrigerant in a constant field instead moving it in and out of magnet, was proposed based on the concept of rotating MCE^12^,^14^. This rotary magnetic refrigerator has certain advantages in comparison with conventional counterpart, such as simple and compact device, and high efficiency. However, compared with polycrystalline materials, the high cost and complexity of preparation for single crystals still hinder the development of this novel magnetic refrigeration technology. To our knowledge, the rotating MCE of polycrystalline materials has never been reported yet. In addition, since the maximum field supplied by a permanent magnet is usually lower than 20 kOe, a large MCE under low field change is desirable for the fulfillment of a magnetic refrigerator simply using permanent magnets. Consequently, it is highly significant to exploit polycrystalline materials with large rotating MCE especially under low field change (i.e., <20 kOe) from both fundamental and practical points of view.

It is known that textured materials with highly preferred crystallographic orientation could also exhibit anisotropic behavior, such as anisotropic magnetostrain along different directions of columnar grains^15^. Thus, a large anisotropy of MCE can be theoretically expected in the materials with both large magnetocrystalline anisotropy and preferred crystallographic orientation. Rare earth-transition metal (*R*–T) intermetallic compounds often behave strong magnetic anisotropy due to the record-high values of magnetic anisotropy constants^7^. In addition, the high magnetic moments of heavy rare earth ions could enhance the change of magnetization between different crystal orientations, and thus resulting in a large rotating MCE^13^. Based on the above analyses, we investigated the rotating MCE in textured DyNiSi compound, which consists of Dy^3+^ ions and presents strong uniaxial magnetocrystalline anisotropy^16^. A giant rotating MCE as well as large conventional MCE were observed in the textured DyNiSi polycrystalline material, suggesting that DyNiSi could be attractive candidate of magnetic refrigerants for both conventional magnetic refrigerator and novel rotary magnetic refrigerator. The present study provides a feasible way to exploit magnetic refrigeration materials with large rotating MCE.

## Results

### Characterization of texture structure

**Figure 1:**
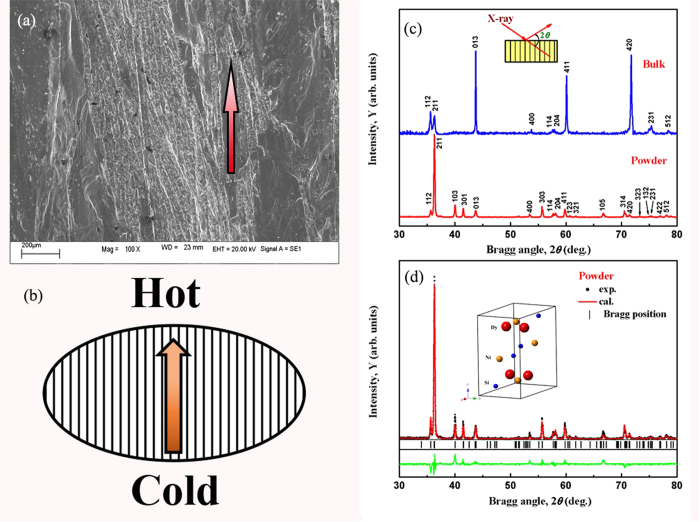
(**a**) The SEM image of fractured DyNiSi button. (**b**) The schematic for the formation of texture structure in DyNiSi compound during arc-melting. (**c**) XRD patterns carried out on DyNiSi powder and on the bulk surface perpendicular to the longitudinal direction of columnar grains. (**d**) The observed (dots) and calculated intensities (line drawn through the data points) of powder XRD patterns at room temperature. The short vertical lines indicate the Bragg peak positions of TiNiSi-type orthorhombic structure. The lower curve shows the difference between the observed and calculated intensities. The inset shows the perspective view of the unit cell of DyNiSi.

Figure 1(a) shows the scanning electron microscopy (SEM) image of fractured DyNiSi button, and it is clearly seen that the sample exhibits a typical texture structure with a series of columnar grains. The formation of texture structure is due to the large temperature gradient between the bottom and top of button during the arc-melting (Fig. 1(b)). In order to further investigate the texture in DyNiSi, both X-ray diffraction (XRD) patterns of powder and bulk samples were detected at ambient temperature as shown in Fig. 1(c). It is noted that the XRD pattern of bulk was taken on the surface of sample which is perpendicular to the crystal orientation of columnar grains. In comparison with powder sample that presents a random crystallographic orientation, many diffraction peaks in bulk XRD pattern become lower while only a few crystal planes, such as (013), (411), and (420), exhibit much stronger intensities, and thus confirming the highly preferred crystallographic orientation^15^,^17^. Figure 1(d) shows the observed and refined powder XRD patterns of DyNiSi compound. The Rietveld refinement reveals that DyNiSi compound crystallizes in a single phase with TiNiSi-type orthorhombic structure (space group *Pnma*). The lattice parameters are determined to be *a *= 6.8540(5), *b *= 4.1524(5), and *c *= 7.1589(6) Å, respectively, which are in a good agreement with the data in previous report^16^.

### Anisotropy of magnetic properties

**Figure 2:**
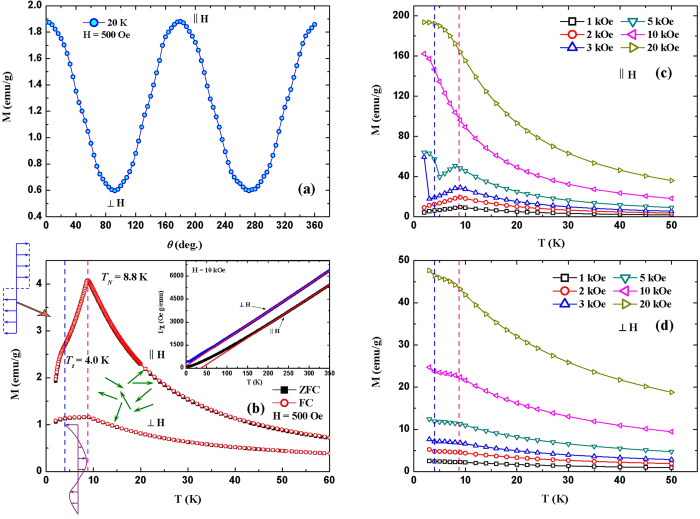
(**a**) The magnetization as a function of rotation angle at 20 K in the magnetic field of 500 Oe. (**b**) Temperature dependence of ZFC and FC magnetization for DyNiSi compound at 500 Oe along the parallel and perpendicular directions, respectively. The inset shows the temperature variation of inverse dc susceptibility (*χ*^−1^) fitted to the Curie-Weiss law in the field of 10 kOe. (**c**) and (**d**) The temperature dependence of magnetization in various magnetic fields along parallel and perpendicular directions, respectively.

Neutron diffraction studies showed that the magnetic moments in *R*NiSi (*R *= Tb, Dy, Ho) are aligned along the *b*-axis, suggesting the strong magnetocrystalline anisotropy^16^. Therefore, considering the highly preferred crystallographic orientation, a large magnetic anisotropy along different crystalline orientation can be expected in this textured DyNiSi compound. The magnetization was measured by rotating the sample in the magnetic field of 500 Oe as shown in Fig. 2(a). Here, the rotation angle *θ* is defined as 0° when the longitudinal direction of columnar grains is parallel to the magnetic field. It is seen that the magnetization decreases gradually by rotating the sample from parallel to perpendicular direction, suggesting that the easy magnetization axis is consistent with the preferred crystalline orientation. Figure 2(b) shows the temperature (*T*) dependence of zero-field-cooling (ZFC) and field-cooling (FC) magnetization (*M*) at 500 Oe along the parallel and perpendicular directions, respectively. It is found that both thermomagnetic curves show similar trend but with different magnetizations. With decreasing temperature, DyNiSi experiences a paramagnetic (PM) to antiferromagnetic (AFM) transition at the Néel temperature *T*_*N*_ of 8.8 K. In addition, another anomaly is observed around the transition temperature *T*_*t*_ = 4 K, which is likely related to the ordering change of magnetic moments from sine to square-modulated structure based on the neutron diffraction studies^16^. It is also noted that no obvious discrepancy between ZFC and FC curves is observed, suggesting the great thermomagnetic reversibility of magnetic transitions. The inverse dc susceptibility (*χ*^−1^) obeys the Curie-Weiss law in the PM region, and then the effective magnetic moments (*μ*_*eff*_) and PM Curie temperatures (*θ*_*P*_), obtained by Curie-Weiss fit of 1/*χ*-*T* curve (inset of Fig. 2(b)), are 10.73 *μ*_*B*_ and 36.2 K for parallel direction, 10.60 *μ*_*B*_ and −8.5 K for perpendicular direction, respectively. Both *μ*_*eff*_ values are close to the theoretical magnetic moment (10.63 *μ*_*B*_) of Dy^3+^ free ion, implying the absence of localized magnetic moment on Ni atoms. However, the considerably large difference of *θ*_*P*_ values indicates the high magnetic anisotropy between different directions^11^. Figure 2(c,d) present the temperature dependence of magnetization in various magnetic fields along different directions. Along parallel direction, an obvious increase of magnetization can be observed below *T*_*t*_ when field is higher than 2 kOe. With further increasing magnetic field, another large jump of magnetization occurs below *T*_*N*_ when field reaches 10 kOe. This result suggests that part of AFM moments below *T*_*t*_ can be easily transformed into ferromagnetic (FM) moments at low fields, and then all moments below *T*_*N*_ order ferromagnetically through another field-induced AFM-FM metamagnetic transition when field reaches 10 kOe. On the other hand, no typical λ-type peak around *T*_*N*_ is observed for all thermomagnetic curves along perpendicular direction, suggesting that a certain amount of FM components may appear below *T*_*N*_. In addition, a weak field-induced metamagnetic transition from AFM to FM states below *T*_*t*_ also occurs at low fields (e.g., *H *> 2 kOe) along perpendicular direction, which is consistent with the result along parallel direction.

**Figure 3:**
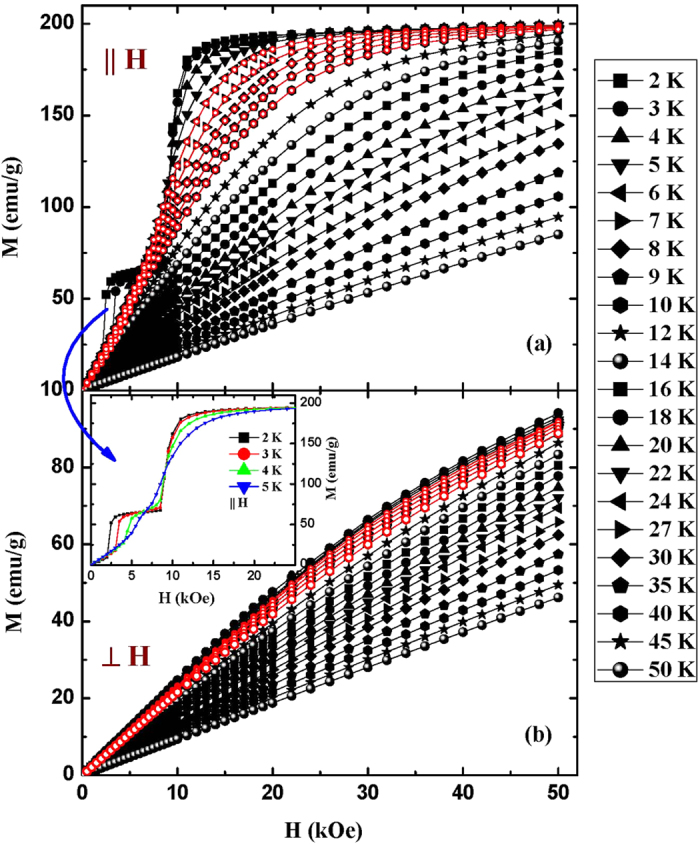
Magnetization isotherms of DyNiSi along parallel (**a**) and perpendicular directions (**b**) respectively. The isotherms with red circles around *T*_*N*_ indicate the *M*-*H* curves measured in field decreasing mode. The inset shows the magnetization isotherms along parallel direction in the temperature range of 2–5 K at low magnetic fields.

Figure 3 shows the magnetization isotherms of DyNiSi along parallel and perpendicular directions, respectively. Here, the magnetic field has been corrected by taking into account the demagnetization effect, and the influence of demagnetization effect on MCE will be discussed later. The magnetization isotherms along parallel direction in the temperature range of 2–5 K at low magnetic fields are displayed in the inset of Fig. 3(b). It is found that the magnetization curves below *T*_*t*_ exhibit two steps with increasing field, corresponding to the successive field-induced AFM-FM metamagnetic transitions with critical fields *H*_*cr*_ = 2 and 8.5 kOe (defined as the maximum of d*M*/d*H* vs. *H* curve), respectively. In the temperature range of *T*_*t*_ − *T*_*N*_, such as *T *= 5 K, only one metamagnetic transition with *H*_*cr*_ = 8.5 kOe can be observed with increasing field. Above result indicates that the sine-modulated magnetic structure is more stable and harder than square-modulated structure to be induced into FM state. Thus, the transition from sine to square-modulated structure may result in some unstable moments, which could be easily induced into FM state at low fields. In addition, the magnetization tends to be saturated at 50 kOe along parallel direction and the saturation magnetization (Μ_*S*_) is determined to be 9.10 *μ*_*B*_/Dy^3+^ by extrapolating 1/*H* to 0 using the *M*–*H* curves at 2 K, slightly lower than the theoretical *gJ* value of 10.0 *μ*_*B*_/Dy^3+^. This fact confirms that the preferred crystal orientation is consistent with the easy magnetization axis. On the other hand, the magnetization along perpendicular direction is much small and does not show a tendency of saturation even at 50 kOe (Fig. 3(b)), revealing the characteristic of hard magnetization axis. Moreover, it is found that the magnetization isotherms show certain curvatures below *T*_*N*_, implying the existence of FM correlations. To investigate the magnetic reversibility, the *M*–*H* curves around *T*_*N*_ were measured in field increasing and decreasing modes. No magnetic hysteresis is found in these isotherms along either parallel or perpendicular directions, indicating the perfect reversibility of magnetic transition which is beneficial to the practical applications of magnetic refrigeration.

### Rotating magnetocaloric effect

**Figure 4:**
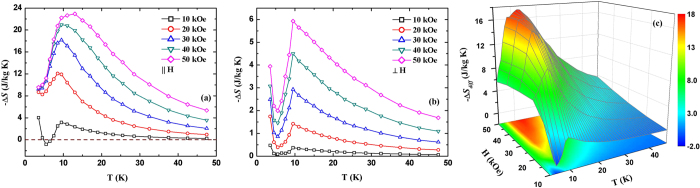
The temperature dependence of Δ*S* for different magnetic field changes along parallel (**a**) and perpendicular (**b**) directions, respectively. (**c**) The difference of Δ*S* between parallel and perpendicular directions as a function of temperature and magnetic field change.

the entropy change Δ*S* can be derived from *M*–*H* curves by using the following equation based on Maxwell relation^10^,^18^:
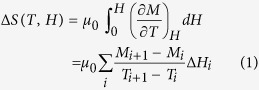


Where *μ*_0_ is the permeability of vacuum, and *M*_*i*_ and *M*_*i*+1_ are the magnetization values measured at temperatures *T*_*i*_ and *T*_*i*+1_ in a magnetic field *H*_i_, respectively. Figure 4(a,b) display the temperature dependence of Δ*S* for different magnetic field changes along parallel and perpendicular directions, respectively. It can be clearly seen that DyNiSi presents a giant anisotropic MCE, e.g., the maximum −Δ*S* values are 22.9 and 5.9 J/kg K around *T*_*N*_ for a field change of 50 kOe along parallel and perpendicular directions, respectively. A small negative −Δ*S* value (inverse MCE) is observed below *T*_*N*_ at 10 kOe along parallel direction because of the presence of AFM state, while it becomes positive with the increase of magnetic field. Such a sign change of −Δ*S* is due to the field-induced AFM-FM metamagnetic transition^19^. In addition, another −Δ*S* peak is found around *T*_*t*_ for both directions. As mentioned before, the transition from sine to square-modulated structure may lead to some unstable moments below *T*_*t*_. Therefore, an applied magnetic field will turn these AFM components into FM ordering which exhibits a magnetically more ordered configuration, and then resulting in a positive −Δ*S* peak. Figure 4(c) shows the difference of Δ*S* between different directions as a function of temperature and magnetic field change. For the field changes of 20 and 50 kOe, the −Δ*S*_*diff*_ peak reaches as high as 11.1 J/kg K at 8.5 K and 17.6 J/kg K at 13 K due to the giant anisotropy of MCE. This result indicates that a large rotating MCE can be obtained by rotating the sample from perpendicular to parallel direction. It should be pointed out that, to our knowledge, this is the first time that large rotating MCE in polycrystalline material has been reported.

The refrigerant capacity (*RC*), a measure of the heat transfer between the hot and cold sinks in an ideal refrigeration cycle, was estimated based on the Δ*S*–*T* curves using the approach suggested by Gschneidner *et al.*^20^:
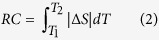


Where *T*_*1*_ and *T*_*2*_ are the temperatures corresponding to both sides of the half-maximum value of –Δ*S* peak, respectively. The *RC* values along parallel direction are obtained to be 133 and 434 J/kg for the field changes of 20 and 50 kOe, respectively. For comparison, the conventional and rotating MCEs of DyNiSi as well as some other refrigerants are listed in Table 1. It can be seen that DyNiSi along parallel direction presents not only larger Δ*S* but also much higher *RC* value in comparison with the conventional MCE of most materials in similar temperature range. In addition, the rotating MCE of DyNiSi between different directions is comparable with or even larger than those of other reported single crystals. Particularly, the rotating Δ*S*_*diff*_ of 11.1 J/kg K for polycrystalline DyNiSi under a low field change of 20 kOe is much higher than those of single crystals with transition temperature around 8.8 K, such as TbMnO_3_ (–Δ*S*_*diff*_ = 2.7 J/kg K at 9 K)^11^ and HoMn_2_O_5_ (–Δ*S*_*diff*_ = 3.2 J/kg K at 10 K)^12^.

**Figure 5:**
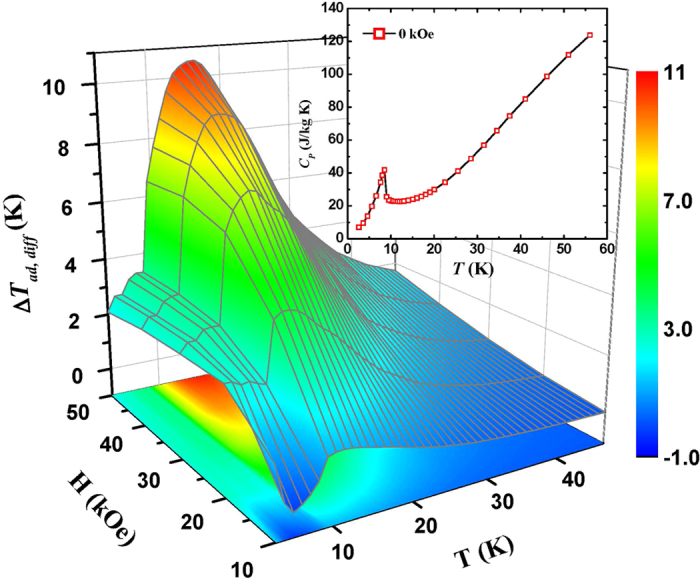
The rotating Δ*T*_*ad*, *diff*_ of DyNiSi between parallel and perpendicular directions as a function of temperature and magnetic field. The inset shows the temperature dependence of heat capacity for DyNiSi in zero field.

As is well known, adiabatic temperature change Δ*T*_*ad*_ has been considered as the most important criterion to evaluate the MCE since it could directly reflect the temperature change of magnetic refrigerants upon the variation in magnetic field^21^. Unfortunately, very few works on rotating Δ*T*_*ad*_ have been reported. Based on the theory of thermodynamics, the Δ*T*_*ad*_ value can be calculated by the following equation:
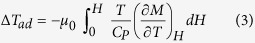


where *C*_*P*_ is specific heat capacity. Considering the insignificant magnetic field dependence of *C*_*P*_ for materials with second order phase transition (SOPT)^22^, the equation (3) can be simply transformed into
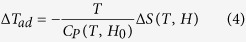


where *C*_*P*_(*T*, *H*_0_) is zero-field heat capacity as shown in the inset of Fig. 5. The typical *λ*-type *C*_*P*_ peak and the absence of thermal and magnetic hysteresis suggest the nature of SOPT for DyNiSi compound. Thus, the rotating Δ*T*_*ad*, *diff*_ of DyNiSi between different directions was calculated by combining *C*_*P*_(*T*, *H*_0_) and Δ*S*_*diff*_(*T*, *H*) values through the equation (4) and is shown in Fig. 5. The maximum values of rotating Δ*T*_*ad*, *diff*_ are 4.4 and 10.5 K under the magnetic fields of 20 and 50 kOe, respectively, which are much larger than those of other rotating magnetic refrigerants reported so far, such as Gd (Δ*T*_*ad*, *diff*_ = 0.3 K at 15 kOe)^23^ and HoMn_2_O_5_ (Δ*T*_*ad*, *diff*_ = 5.2 K at 50 kOe)^12^. Very recently, Balli *et al.* proposed a rotary magnetic refrigerator in which a regenerator constituted of HoMn_2_O_5_ single crystal blocks is rotated in a constant magnetic field, and then the liquefaction of helium and hydrogen could be realized by using them as heat transfer fluids^12^. Compared with single crystal HoMn_2_O_5_, the larger rotating MCE and easier preparation process make textured DyNiSi polycrystalline material attractive candidate of magnetic refrigerants for this novel rotary magnetic refrigerator, which could be applied in the liquefaction of helium and hydrogen.

**Figure 6:**
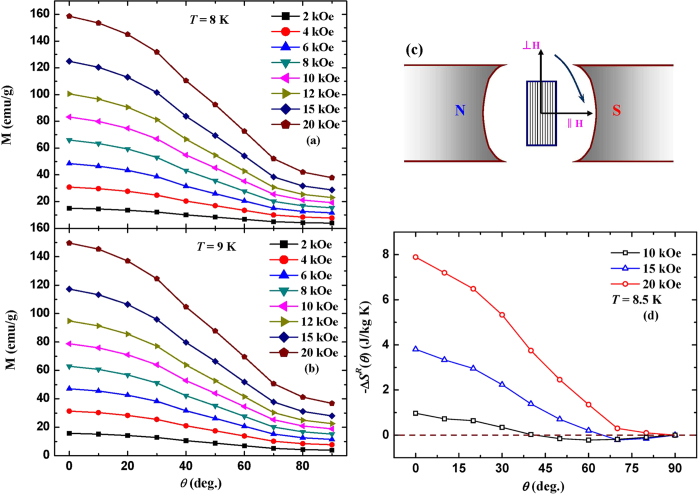
Isothermal magnetization curves at 8 K (**a**) and 9 K (**b**) under applied fields up to 20 kOe by rotating the sample from perpendicular (90°) to parallel (0°) direction with a step of 10°. (**c**) The schematic describes the rotation of sample from perpendicular (90°) to parallel (0°) direction in magnetic field. (**d**) The Δ*S*^*R*^(*θ*) as a function of rotation angle at 8.5 K for different magnetic field changes.

In order to further investigate the rotating MCE, the isothermal magnetization curves at 8 and 9 K were measured under applied fields up to 20 kOe by rotating the sample from perpendicular (90°) to parallel (0°) direction with a step of 10° as shown in Fig. 6. It has to be pointed out that the demagnetization correction has not been performed due to the difficult determination of demagnetization factor in different directions. By defining the rotating entropy change Δ*S*^*R*^(90°) as zero, the Δ*S*^*R*^(*θ*) value at 8.5 K can be obtained based on the magnetization curves in Fig. 6(a,b) by using the following equation:
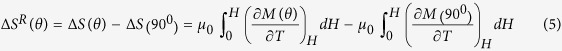


Figure 6(d) shows the Δ*S*^*R*^(*θ*) as a function of rotation angle for different magnetic field changes. It is worth noting that small negative −Δ*S*^*R*^(*θ*) value is observed under relatively low fields near the perpendicular direction. This can be understood that the disordering of magnetic moments in AFM sublattice is enhanced under low fields when the rotation just starts, and thus it results in the positive entropy change. With further rotating the sample towards parallel direction, the majority of spins in AFM sublattice would orient along the field direction, which consequently increases the spin ordering and gives rise to the positive −Δ*S*^*R*^(*θ*) value. Under the magnetic field of 20 kOe, the −Δ*S*^*R*^(*θ*) value increases gradually and reaches a maximum of 7.9 J/kg K as the sample is rotated from perpendicular to parallel direction.

## Discussion

As mentioned before, the large rotating MCE in single crystal materials is mainly related to high magnetocrystalline anisotropy^7^,^11^ as well as large magnetic moments of rare earth ions^13^. Similarly, the large rotating MCE in polycrystalline DyNiSi compound can be attributed to the following key factors: First, the strong uniaxial magnetocrystalline anisotropy in DyNiSi compound is the intrinsic origin of large rotating MCE. Second, highly textured structure keeps the strong magnetocrystalline anisotropy in polycrystalline DyNiSi compound. Third, high saturation moments of Dy^3+^ ions result in the large change of magnetization between different crystal orientations. Based on above discussion, we believe that, from the viewpoint of material design, large rotating MCE can be also obtained in other polycrystalline materials if above conditions can be satisfied. Considering the high cost and complexity of preparation for single crystals, our result suggests that textured polycrystalline materials may be better candidates of magnetic refrigerants for rotary magnetic refrigerator.

It should be pointed out that the large magnetic anisotropy in DyNiSi includes not only intrinsic magnetocrystalline anisotropy, but also extrinsic shape anisotropy that is related to the demagnetization effect. Previous studies have demonstrated that the demagnetization effect is considerably large and cannot be ignored in the determination of true MCE^24^,^25^. Assuming the internal field *H*_*int*_ is homogeneous across the non-ellipsoidal sample, the average internal field can be estimated by subtracting the demagnetization field *H*_*d*_ = *N*_*d*_*M* from the external field *H*_*ext*_,
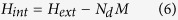


where *N*_*d*_ is the demagnetization factor and can be calculated according to the model in Ref. [26]. Here, the demagnetization factors *N*_*d*_ are obtained to be 0.1983 and 0.4032 for parallel and perpendicular directions, respectively. It is seen from Fig. 4 that DyNiSi exhibits a giant anisotropic MCE after demagnetization correction, suggesting that the large anisotropy of MCE is mainly due to the strong magnetocrystalline anisotropy. In addition, it is found that the –Δ*S*^*R*^(0°) value without demagnetization correction is 7.9 J/kg K at *H*_*ext*_ = 20 kOe, which is lower than the –Δ*S*_*diff*_ value with demagnetization correction (11.1 J/kg K at *H*_*int*_ = 20 kOe). This fact indicates that the demagnetization effect would result in an underestimation of real MCE (this is also confirmed by Supplementary Fig. S1).

In conclusion, we have demonstrated that textured DyNiSi exhibits giant anisotropy of MCE. A large reversible Δ*S* as well as high *RC* have been observed along parallel direction, which are much larger than those along perpendicular direction. Therefore, a giant rotating MCE was obtained by rotating the sample from perpendicular to parallel direction. To our knowledge, this is the first time that rotating MCE has been reported in polycrystalline materials. This result suggests that textured DyNiSi polycrystalline material is not only a good candidate of magnetic refrigerants for conventional magnetic refrigeration, but also a promising material for novel rotary magnetic refrigeration which could have more simplified and efficient magnetic cooling systems. From the viewpoint of material design, we believe that three conditions must be satisfied to obtain large rotating MCE in polycrystalline materials: (1) strong magnetocrystalline anisotropy which is the intrinsic origin of large rotating MCE, (2) highly preferred crystallographic orientation which could ensure the existence of strong magnetocrystalline anisotropy in polycrystalline materials, and (3) large difference of magnetization between different crystal orientations. Consequently, present study opens a new way to exploit magnetic refrigeration materials with large rotating MCE.

## Methods

The polycrystalline DyNiSi compound was synthesized by arc-melting appropriate proportion of constituent components with the purity better than 99.9 wt.% in a water-cooled copper hearth under purified argon atmosphere. The as-cast sample was annealed in a high-vacuum quartz tube at 1073 K for 7 days, and then quenched into liquid nitrogen. The X-ray diffraction (XRD) measurements on bulk and powder samples were performed at room temperature by using Cu *Kα* radiation. The Rietveld refinement based on the powder XRD pattern confirms that the annealed sample crystallizes in a single phase with TiNiSi-type orthorhombic structure (space group *Pnma*). Microstructures were investigated by scanning electron microscopy (SEM) using a LEO-1450 microscope. A rectangular-like sample of 1.2 mg with dimensions 1.0 mm × 0.5 mm × 0.5 mm was selected for the magnetic measurements. Magnetization was measured using a commercial superconducting quantum interference device (SQUID) magnetometer from Quantum Design Inc (model MPMS-XL). The magnetization as a function of rotation angle was measured under applied fields up to 20 kOe by rotating the sample from perpendicular to parallel direction with a step of 10°. Here, the rotation angle *θ* is defined as 0° when the longitudinal direction of columnar grains is parallel to the magnetic field.

## Additional Information

**How to cite this article**: Zhang, H. *et al.* Giant rotating magnetocaloric effect induced by highly texturing in polycrystalline DyNiSi compound. *Sci. Rep.* **5**, 11929; doi: 10.1038/srep11929 (2015).

## Supplementary Material

Supplementary Information

## Figures and Tables

**Table 1:** Conventional and rotating magnetocaloric properties for DyNiSi and some other refrigerant materials.

Materials	*T_ord_ (K)*		**−Δ*S* (J/kg K)**		***RC* (J/kg)**	Refs
			**20 kOe**	**50 kOe**	**50 kOe**	
DyNi_2_B_2_C	10.5		4.4	17.6	186^a^	27
HoCoAl	10		12.5	21.5	437^a^	28
Dy_0.9_Tm_0.1_Ni_2_B_2_C	9.2		4.05	14.7	220^a^	29
ErNi_5_	9		−	15.0	232^a^	30
ErNiIn	9		10.1	15.1	229	31
Single crystal PrSi	52	90° rotationfrom *a* to *b* axis	10.8	18.6	240^b^	32
Single crystal DyFeO_3_	4.5	90° rotationfrom *c* to *b* axis	16.6	19.5	391	13
Single crystal ErFeO_3_	4.1	90° rotationfrom *b* to *c* axis	11.9	13.5	234^b^	33
Single crystal TmMnO_3_	16	90° rotationfrom *a* to *c* axis	0.12	5.25	100^b^	34
Single crystal TbMnO_3_	9	90° rotation from *b* to *a* axis	2.7	11.5	201^b^	11
Single crystal HoMn_2_O_5_	10	90° rotationfrom *c* to *b* axis	3.2	10.0	198^b^	12
Textured DyNiSi		Parallel	12.1	22.9	434	
	8.8	Perpendicular	1.4	5.9	99	This work
		90° rotation	11.1	17.6	328^b^	

^a^The *RC* values were estimated from the Δ*S*–*T* curves in the reference literatures.

^b^The *RC*_*diff*_ values were estimated from the Δ*S*_*diff*_ –*T* curves.
