# Supplementary material for: Giant rotating magnetocaloric effect induced by highly texturing in polycrystalline DyNiSi compound
Source: Sci Rep. 2015 Jul 10;5:11929. doi: 10.1038/srep11929 (PMC5155619; doi:10.1038/srep11929)
Supplement: Supplementary Information [file srep11929-s1.pdf]

Giant rotating magnetocaloric effect induced by highly texturing in polycrystalline DyNiSi compound

Hu Zhang,[1, *] YaWei Li,[1] Enke Liu,[2] YaJiao Ke,[2] JinLing Jin,[2] Yi Long,[1] and BaoGen Shen[2]

[1]School of Materials Science and Engineering, University of Science and Technology of Beijing, Beijing 100083, P R China.

[2]State Key Laboratory for Magnetism, Institute of Physics, Chinese Academy of Sciences, Beijing 100190, P R China.

*zhanghu@ustb.edu.cn

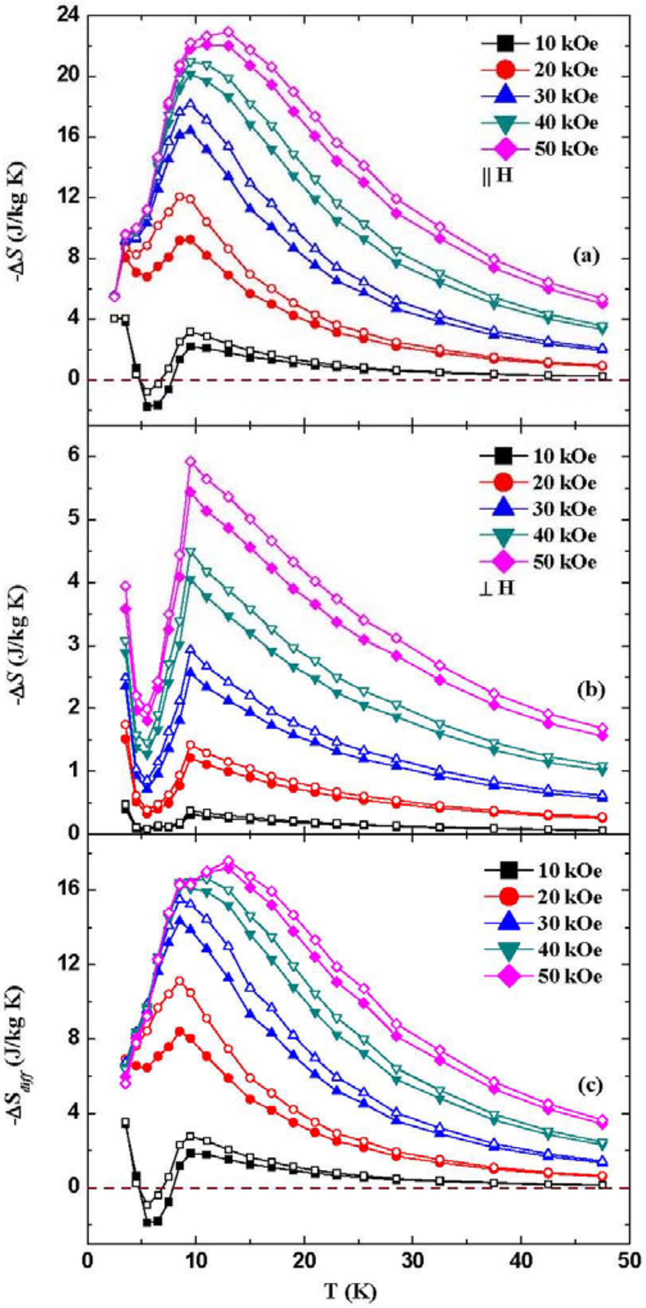

Figure S1 | The temperature dependence of Δ*S* with (open symbols) and without (full symbols) demagnetization correction for different magnetic field changes along parallel (a) and perpendicular (b) directions, respectively, and (c) the difference of Δ*S* between parallel and perpendicular directions as a function of temperature.
